# Non‐clinical Efficacy and Safety of SUVN‐L3307032, a Muscarinic M4 Positive Allosteric Modulator (M4 PAM), for the Treatment of Neuropsychiatric Symptoms.

**DOI:** 10.1002/alz70859_100081

**Published:** 2025-12-25

**Authors:** Renny Abraham, Venkatesh Goura, Rajesh Kallepalli, Rajesh Babu Medapati, Onamala Varalakshmi, Kumar Bojja, Aamer Shaikh, Rajesh Kumar Badange, Sravanthi Manchineella, Tirumala Narasimhula, Ankit Das, Naga Sai Soma Chandra Sekhar Guduru, Keerthi Sai Bitra, Kavya Sree Mukkollu, Mary Praveena Nissankarao, Santosh Kumar Pandey, Vinod Kumar Goyal, Ramakrishna Nirogi

**Affiliations:** ^1^ Suven Life Sciences Ltd, Hyderabad, Telangana India

## Abstract

**Background:**

Neuropsychiatric symptoms (NPS) associated with Alzheimer’s disease (AD) are burdensome to the caregiver and patients. The current therapies for the treatment of NPS in AD are associated with limited efficacy and severe side effects. Hence, newer treatment options are required to effectively control these symptoms. The combination of xanomeline and trospium (KarXT) has recently been approved for the treatment of schizophrenia and has been proposed for the treatment of NPS associated with AD as well. Xanomeline, a muscarinic M1/M4 agonist, is likely to induce peripheral side effects. To overcome peripheral side effects, trospium, a peripheral anticholinergic agent, is administered with xanomeline. Since positive allosteric modulators (PAMs) act on the allosteric site rather than the orthosteric site, they are less likely to induce peripheral side effects. Here, we present the safety and pharmacodynamics properties of an M4 PAM, SUVN‐L3307032.

**Method:**

The in‐vitro properties of SUVN‐L3307032 were characterized. Its effect on the allosteric site of the muscarinic M4 receptors was characterized using reporter gene assay. The efficacy of SUVN‐L3307032 was assessed in an amphetamine induced hyperlocomotion test using an open field. Receptor occupancy of SUVN‐L3307032 was assessed using non‐radiolabeled based method. SUVN‐L3307032 was assessed for its efficacy to reverse hallucinations induced by 2,5‐dimethoxy‐4‐iodoamphetamine hydrochloride (DOI) by assessing head twitch responses. Efficacy of SUVN‐L3307032 in reversing cognitive deficits associated with psychiatric symptoms was assessed in a fear conditioning task. Preliminary toxicity studies were conducted in rats and dogs to evaluate the safety of SUVN‐L3307032.

**Result:**

SUVN‐L3307032 was found to be a selective M4‐PAM. SUVN‐L3307032 attenuated amphetamine induced hyperlocomotion in an open field test. In the fear conditioning task, SUVN‐L3307032 reversed amphetamine induced cognitive deficits. SUVN‐L3307032 attenuated head twitch responses induced by DOI. The observations from the amphetamine‐induced hyperlocomotion assay correlated well with the occupancy at the allosteric site of muscarinic M4 receptors. Preliminary toxicity studies did not show any concerns for further development.

**Conclusion:**

SUVN‐L3307032 has the potential to be a new therapeutic option for the treatment of NPS associated with AD.

